# Protection of Liver as a Remote Organ after Renal Ischemia-Reperfusion Injury by Renal Ischemic Postconditioning

**DOI:** 10.1155/2014/120391

**Published:** 2014-03-12

**Authors:** Behjat Seifi, Mehri Kadkhodaee, Atefeh Najafi, Atefeh Mahmoudi

**Affiliations:** ^1^Department of Physiology, Faculty of Medicine, Tehran University of Medical Sciences, Tehran 14155-6447, Iran; ^2^Department of Anatomy, Faculty of Medicine, Tehran University of Medical Sciences, Tehran 14155-6447, Iran

## Abstract

This study was designed to investigate the protective effects of local renal ischemic postconditioning (POC) on liver damage after renal ischemia-reperfusion (IR) injury. Male rats were divided into three groups  (*n* = 8). They underwent a right nephrectomy before induction of 45 minutes of left kidney ischemia or sham operation. POC was performed by four cycles of 10 seconds of ischemia and 10 seconds of reperfusion just at the beginning of 24 hours of reperfusion. Then blood and liver samples were collected to measure serum aspartate aminotransferase (AST), alanine aminotransferase (ALT), and liver oxidative stress parameters including superoxide dismutase (SOD) activity and malondialdehyde (MDA) level. Renal IR caused a significant increase in liver functional indices as demonstrated by increased serum AST and ALT compared to sham group. These parameters reduced significantly in POC group compared to IR group. Liver MDA levels increased and SOD activity decreased in IR group compared to sham group. Induction of POC reduced the elevated liver MDA levels and increased the reduced liver SOD activity. These results revealed that renal IR injury causes liver damage as a remote organ and POC protects liver from renal IR injury by a modification in the hepatic oxidative stress status.

## 1. Introduction

Acute kidney injury (AKI) is a common complication that occurs in some of the hospitalized patients especially in intensive care units. Renal ischemia reperfusion (IR) is one of the most important causative mechanisms of AKI [1]. High mortality rate during AKI is largely due to the remote organ dysfunction. Renal IR injury may also lead to the failure of other systems like lung, brain, and liver [1, 2]. 

Humoral or cellular factors are thought to be the causes of remote organ failure, but their exact pathophysiological mechanisms are not completely understood [3, 4]. Previous studies have shown that AKI causes an increase in leukocyte infiltration in remote organs such as the liver. It has also been demonstrated that renal IR induces oxidative stress in the liver resulting in hepatic dysfunction. Renal IR caused an increase in hepatic tumor necrosis factor levels, myeloperoxidase activities, and thiobarbituric acid reactive substance (TBARS) concentrations [5, 6]. During renal IR, liver functional indices such as blood AST and ALT were elevated and spermine-spermidine acetyl transferase, an enzyme upregulated in early phases of hepatic injury, was increased [2].

Superoxide dismutase (SOD) and catalase are among the most important enzymatic antioxidant systems in the body. SOD, as the first and most important line of defense against reactive oxygen metabolites, transforms superoxide ion to H_2_O_2_ that is a less reactive molecule [7]. Reactive oxygen species degrade polyunsaturated fatty acids, forming MDA, a cytotoxic reactive aldehyde which can be used as a biomarker to measure the level of oxidative stress in an organism [8].

In recent years, ischemic conditioning (i.e., brief intermittent episodes of ischemia) has been considered to protect against prolonged lethal ischemia. More recently, postconditioning (POC, intermittent interruptions of blood flow at the beginning of reperfusion) has been discussed as a more practical approach than ischemic preconditioning (IPC). Similar to preconditioning, POC triggers signaling pathways and activates effectors implicated in other cardioprotective maneuvers. However, the detailed mechanisms underlying these actions are unknown. It was suggested that POC reduces the cardial reperfusion-induced injury, blunts oxidant mediated damages, and attenuates the local inflammatory response to reperfusion [9].

This study was designed to investigate the protective effects of renal ischemic POC on liver damage after renal IR injury. The possible role of POC in reduction of IR-induced oxidative stress in the liver was also investigated.

## 2. Methods

After right nephrectomy, twenty-four male Sprague-Dawley rats (250 to 300 g) were randomly divided into three groups, eight in each: sham, IR, and POC groups. Rats were anaesthetized by intraperitoneal injection of sodium pentobarbital (60 mg/kg Sigma-Aldrich, Steinheim, Germany). Body temperature was maintained at 37 ± 1°C. Systolic blood pressure was measured by the tail-cuff method connected to a pressure transducer (MLT 0380, ADInstruments, Castle Hill, Australia). In the IR group, 45 min of left renal artery occlusion was induced followed by 24 hours of reperfusion. In the sham group, all of the above surgical procedures were applied without induction of IR. In POC group, after induction of 45 min ischemia, 4 cycles of 10 seconds of ischemia and 10 seconds of reperfusion were applied to the kidney just at the beginning of 24 hours of reperfusion to the kidney. After 24 hours, serum and liver tissue samples were collected for measuring of hepatic functional indices and assessment of hepatic oxidative stress status. Blood samples were centrifuged at 4000 g for 10 min at 4°C, and serum was collected for biochemical analysis. Liver tissues were washed in cold phosphate-buffered saline and snap-frozen in liquid nitrogen. The samples were stored at −70°C until further study.

*Biochemical Assay.* Serum aspartate aminotransferase (AST) and alanine aminotransferase (ALT) were used as hepatic functional indices and were measured by commercially available kits. 

*Measurement of Hepatic Oxidative Stress Markers.* The tissue MDA level was determined by the method of Esterbauer and Cheeseman [10] based on its reaction with thiobarbituric acid at 90–100 C and measurement of the absorbance at 532 nm. MDA reacts with thiobarbituric acid (TBA) and produces a pink pigment which has a maximum absorption at 532 nm. The value of each sample was obtained from the standard curve and was expressed as *μ*mol/g tissue. SOD activity was measured according to the Paoletti and Mocali method [11]. In this assay, superoxide anion is generated from molecular oxygen in the presence of EDTA, manganese (II) chloride, and mercaptoethanol. Nicotinamide adenine dinucleotide phosphate oxidation is linked to the availability of superoxide anions in the medium. 

*Statistical Analysis.* Data are expressed as the mean ± SEM. Comparisons among groups were made by one-way ANOVA followed by Tukey test. *P* < 0.05 was considered statistically significant.

## 3. Results

### 3.1. Effect of POC on Renal IR-Induced Hepatic Dysfunction

**Figure 1:**
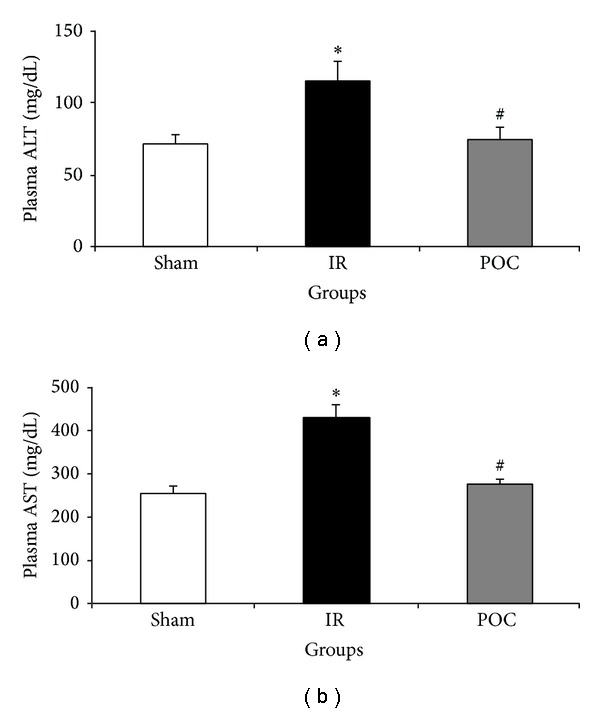
Changes in serum ALT (a) and AST (b) in different groups. The data are presented as mean ± SEM. *P < 0.05 versus sham group. ^#^ *P* < 0.05 versus IR group. IR: 45 min renal ischemia followed by 24 hours of reperfusion. POC group: 4 cycles of 10 seconds of intermittent IR just at the beginning of reperfusion in the kidney.

Serum levels of ALT and AST, hepatic functional parameters, were significantly increased in IR group compared to the sham group (114.5 ± 13.73 versus 70.62 ± 7.75 mg/dL and 428.4 ± 31.39 versus 253.28 ± 18.13 mg/dL, *P* < 0.05, Figure 1). These indices were significantly lower in POC group compared to the IR group (73.71 ± 8.94 versus 114.5 ± 13.73 mg/dL and 274.4 ± 31.33 versus 428.4 ± 31.39 mg/dL, *P* < 0.05, Figure 1).

### 3.2. Effect of POC on Renal IR-Induced Hepatic Oxidative Stress Indices

**Figure 2:**
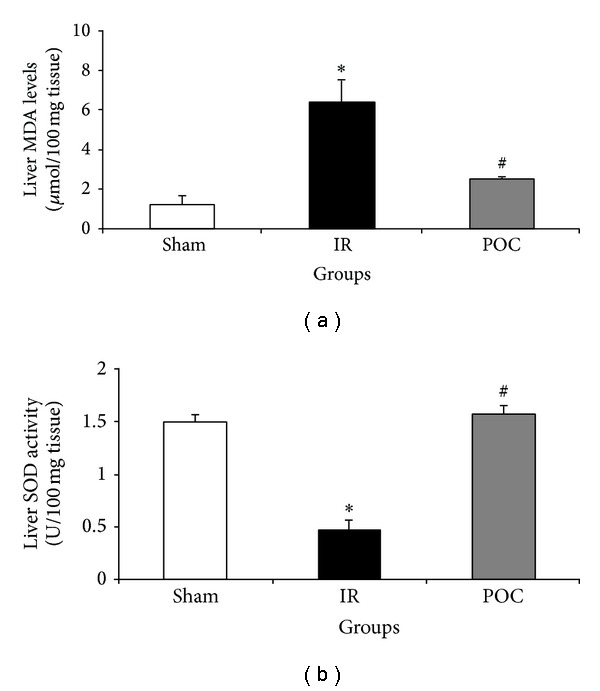
Changes in liver MDA levels (a) and SOD activity (b) in different groups. The data are presented as mean ± SEM. *P < 0.05 versus sham group. ^#^ *P* < 0.05 versus IR group. IR: 45 min renal ischemia followed by 24 hours of reperfusion. POC group: 4 cycles of 10 seconds of intermittent IR just at the beginning of reperfusion in the kidney.

Hepatic MDA levels were increased and SOD activity was decreased in IR group compared to the sham group (6.31 ± 1.16 versus 1.24 ± 0.42 *μ*mol/100 mg tissue and 0.46 ± 0.1 versus 1.48 ± 0.08 U/100 mg tissue, *P* < 0.05, Figure 2). Induction of renal POC prevented the IR-induced reduction in hepatic SOD activity and IR-induced increase in hepatic MDA level (2.48 ± 0.15 versus 6.31 ± 1.16 *μ*mol/100 mg tissue and 1.55 ± 0.08 versus 0.46 ± 0.1 U/100 mg tissue, *P* < 0.05, Figure 2).

## 4. Discussion

The present study demonstrated that renal IR leads to the damage to the liver as a remote organ. Our findings suggest that renal POC attenuates IR-induced liver functional injury and MDA levels and increases SOD activity in liver tissues compared to the IR group.

Several mechanisms are suggested to be involved in remote organ failure, but their exact pathophysiological roles are not completely understood [3, 4]. Chemokines and mitochondrial products activate neutrophils to amplify remote lung injury during mouse acute liver failure [12]. Renal IR and bilateral nephrectomy result in uncontrolled expression of interleukin-17A in the small intestines [13]. IL-17A is a proinflammatory cytokine that causes recruiting neutrophils, activates T cells, and induces expression of other cytokines and chemokines such as TNF-*α* and IL-6 in liver tissue. Increased oxidative stress and production of reactive oxygen species in the liver that were shown in the present study are also thought to play a key role in triggering and maintaining the inflammatory response. Malondialdehyde, an index of lipid peroxidation, was found to be increased in the liver after both renal ischemia reperfusion and bilateral nephrectomy [14]. In addition, hepatic glutathione, an important endogenous free radical scavenger with protective effects on the liver, was decreased. Administration of glutathione before renal IR decreased histological evidence of liver injury and malondialdehyde concentrations and reduced transaminitis [2]. In addition, renal IR leads to decreased concentrations of antioxidant enzymes including myeloperoxidase, superoxide dismutase, and catalase [6]. In the present study, changes in AST and ALT in serum following renal IR are indicators of remote organ failure in liver. Induction of oxidative stress and the increased production of reactive oxygen species, as a result of renal reperfusion injury, are thought to play key roles in triggering the damage locally or to the remote organs. Oxidative stress is suggested to be involved in triggering and maintaining the inflammatory response. This is the mechanism that has been addressed before by us and others.

The increasing mortality rate in patients with AKI necessitates the design of strategies to better understand and assess the impact of kidney injury on distant organs. There is abundant evidence that the sudden restoration of blood flow to ischemic tissue may paradoxically exaggerate injury that is not present at the end of ischemia and could be modified by interventions given only at reperfusion [15]. Reperfusion elicits a wide range of injuries depending on the timing of restoration of blood flow and involves a number of triggers, mediators, and end effectors that are responsible for vascular endothelial dysfunction, upregulation of adhesion molecules on the endothelium, transendothelial emigration of inflammatory cells, tissue edema, infarction, and apoptosis. Therefore, it is valuable to explore clinically applicable and effective therapeutic strategies to reduce postischemic injury. POC, intermittent interruptions of blood flow at the beginning of reperfusion, has been discussed as a practical approach that can trigger signaling pathways and activate effectors. One promising finding of the present study was that induction of remote POC resulted in the protection of liver tissue, demonstrated by reduction in ALT and AST. Data from this study also showed that POC protected the liver from renal IR injury by a modification in the hepatic SOD activity and MDA level. Thus, it can be concluded that the changes in hepatic oxidative stress markers and liver functional indices are in harmony. In other words, in those groups of rats where the oxidative stress is higher, the liver is less functional. Penna et al. suggested that POC reduces the cardiac reperfusion-induced injury, blunts oxidant mediated damages, and attenuates the local inflammatory response to reperfusion [9]. In a study by Liu et al., POC protection is evidenced by increase in nitric oxide release and NO synthase expression [16]. In another study, it was found that POC with a consecutive sequence of 3, 6, and 12 min of reperfusion separated by 5 min of reocclusion reduced creatinine and BUN levels. Protection was associated with preservation of mitochondrial function after 24 and 48 hours of reperfusion [17]. Furthermore, attenuation of reperfusion injury with POC in the kidney has also been associated with phosphorylation of Akt and ERK1/2 [18] and preservation of antioxidant enzymes such as superoxide dismutase, catalase, and glutathione peroxides [19]. Eldaif et al. suggested that attenuation of renal ischemia reperfusion injury by POC involves adenosine receptors and PKC activation [20]. In the present study, POC protected the liver, as a remote organ, from renal IR injury by reductions in the oxidative stress markers and preservation of the antioxidative enzymes. The present study is compatible with the past reports in which it has been demonstrated that POC was able to decrease the systemic damage intensity after a small intestinal ischemic-reperfusion episode [21].

In conclusion, this study suggests that AKI initiates distant organ dysfunction and POC protects the liver as a remote organ from the renal ischemia reperfusion injury. These beneficial effects of POC may be due to the reduction of oxidative stress parameters.
